# Meta-analysis of diagnostic test accuracy studies with multiple thresholds for data integration

**DOI:** 10.4178/epih.e2022083

**Published:** 2022-09-28

**Authors:** Sung Ryul Shim

**Affiliations:** Department of Health and Medical Informatics, Kyungnam University College of Health Sciences, Changwon, Korea

**Keywords:** Evidence based medicine, Meta-analysis, Systematic review, Diagnostic tests, Diagnostic test accuracy, Threshold

## Abstract

**OBJECTIVES:**

The objective of this study is to introduce methods to use all of the information without omission when individual studies provide multiple effect sizes according to multiple cut-off values (thresholds) during diagnostic test accuracy (DTA) for data integration. For diagnostic test meta-analysis, a general performance method for synthesizing data according to one cut value in one study and a performance method for synthesizing data according to two or more cut values in one study were compared and analyzed.

**METHODS:**

As sample data for meta-analysis of DTA studies, 13 DTA studies on prostate cancer (34 effect sizes including total cut-offs) were collected. The summary statistics were calculated and the summary line was analyzed using the “meta”, “mada”, and “diagmeta” packagesof the R software.

**RESULTS:**

The summary statistics of the random effect model univariate analysis of the “meta” package with a single cut-off corresponding to the highest Youden index in a single study and those of the bivariate analysis of the “mada” package were highly similar. However, in the bivariate analysis of the “diagmeta” package including all cut-off values, the sensitivity decreased and the specificity increased as the amount of data increased.

**CONCLUSIONS:**

Considering the heterogeneity of the summary receiver op erating characteristic curve and the use of all given cut-offs, the use of the bivariate analysis model of the “diagmeta” package is recommended. This study focused on practical methods of DTA rather than theoretical concepts for use by researchers whose fields of study are non-statistics related. By performing this study, we hope that many researchers will use R software to determine the DTA more easily, and that there will be greater interest in related research.

## INTRODUCTION

In this paper, we present a methodology for meta-analysis of diagnostic test accuracy (DTA) studies when the individual studies have multiple effect sizes according to multiple cut-off values (thresholds). The proposed method allows the use of all the information without omission in cases of multiple thresholds and multiple effect sizes. Therefore, this study requires basic understanding of meta-analysis of DTA studies as well as prior knowledge in relation to statistical models for calculation of summary statistics by referring to existing works [1-4].

In the case of general pairwise meta-analysis, one effect size is calculated, whereas in meta-analysis of DTA studies, two paired effect sizes are combined simultaneously; for example, the estimates of sensitivity and specificity are calculated at the same time. In the meta-analysis of DTA studies, it is assumed that the cut-off values (thresholds) of the target studies are similar; however, in practice, the cut-off values are not identical, which may be an important risk factor of heterogeneity in the summary receiver operating characteristic (SROC) curve [1].

A number of existing systematic reviews have investigated the methods for calculating effect sizes according to multiple thresholds in meta-analysis of DTA studies [5-9]. In these prior studies, the Youden index has been used, in which a point where the sum of the specificity and sensitivity has the maximum value is derived through the simple formula of “sensitivity+specificity−1.” In the receiver operating characteristic (ROC) curve of a single study, a single cut-off value, the highest, is selected and a bivariate model is used, whereas the cut-off values that are not selected are discarded. In this way, although there are multiple effect sizes for multiple cut-off values, only one effect size is selected. In this case, incomplete reporting occurs by choice, which is a contradictory consequence. Thus, it is not possible to use all the available information.

To address this problem, previous research works on meta-analysis of DTA studies have used all the effect sizes according to multiple cut-off values [10-17]. We introduce a method for estimating the optimal cut-off value, sensitivity, and specificity, which is based on the total summary statistics and the SROC curve by using the distribution functions of the diseased and non-diseased groups, respectively [17].

## MATERIALS AND METHODS

### Diagnostic test summary statistics and models

DTA studies are typically represented by summary statistics and a summary line of a basic 2× 2 table consisting of true positive (TP), false positive (FP), false negative (FN), and true negative (TN) values. In general, representative summary statistics include sensitivity, specificity, positive predictive value (PPV), and negative predictive value (NPV). An example of a summary line for summary statistics is the SROC curve.

For calculation of the summary statistics of DTA studies, an appropriate model needs to be selected, as in the case of the general pairwise meta-analysis. Models with simultaneous consideration of sensitivity and specificity include the bivariate and hierarchical models.

In the bivariate model, a binominal distribution that directly models the sensitivity and specificity is assumed for within-study variations and a bivariate normal distribution is assumed for between-study variations. The hierarchical model assumes a binominal distribution for within-study variations; for between-study variations, a hierarchical distribution is assumed for the parameters included in the logistic model by applying the logistic regression model to determine the probability of a binominal distribution [1,4]. However, both models are similar to the random effect model of a general pairwise meta-analysis, and the two models produce the same value mathematically when there is no covariate [18,19].

In this study, estimation of the values will be performed using the “meta”, “mada”, and “diagmeta” packages of the R software, and these packages need to be installed before running the software.

• install.packages(“meta”)

• install.packages(“mada”)

• install.packages(“diagmeta”)

### Data coding and loading

As sample data for meta-analysis of DTA studies (Table 1), 13 DTA studies on prostate cancer (34 effect sizes including total cut-offs) were collected. In the table, the biomarkers indicate diagnostic markers for each test, and the cut-off indicates the threshold. The Youden index was calculated according to the formula of “sensitivity+specificity−1”, and if there were two or more cut-offs within the same study, the variable of Youden index choice (Y.c) value corresponding to the highest cut-off was coded as 1.

In univariate and bivariate analyses using meta and mada functions, one threshold with the highest Youden index was selected in a single study for analysis. In bivariate analysis using the diagmeta function, all thresholds were included in the analysis and the optimal threshold was estimated.

### Ethics statement

This study used publicly available data and did not include human participant research. Therefore, this study was not submitted for institutional review board approval and did not require informed consent procedures.

## RESULTS

### Univariate analysis

The “metaprop” function from the “meta” package calculates the total effect size when there are a number of events (event) and a number of samples (n) in the proportion-type data.

In sensitivity analysis, the number of events is TP and the number of samples (n) is TP+FN. Among the methods for calculating the effect size in proportion-type data, the method based on logit transformation and then back transform was used (the logit transformation and Clopper–Pearson method). When the assumptions of the statistical model are properly applied for consistency and considering the symmetry and distribution of the data, it is preferable to transform (log transformation or logit transformation) the proportion-type data because this produces conservative results through the transformation. The inverse variance method is a basic method of meta-analysis, which utilizes the inverse variance of the applicable study when calculating the weights of individual studies, and the tau value, which is the between-study variance, was calculated using the DerSimonian–Lair estimator in a random effect model.

**Figure 1.**
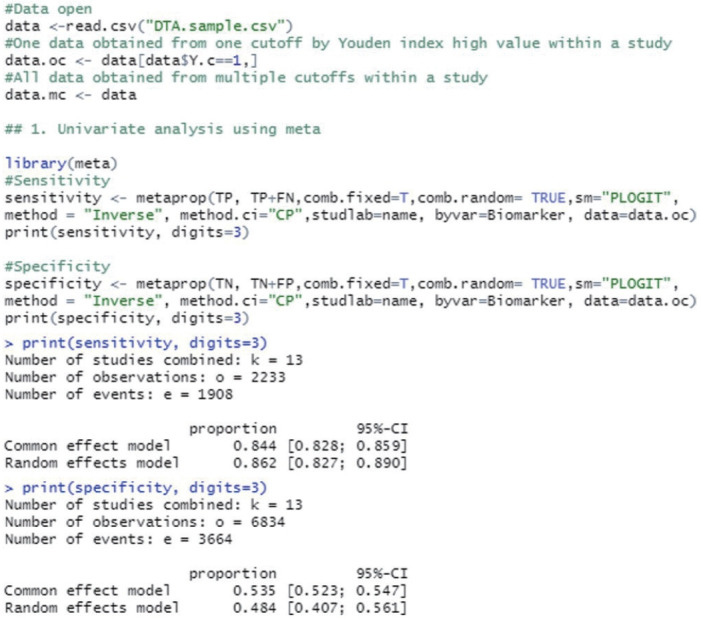
Univariate analysis using meta package.

The sensitivity was 0.844 in the common effect model and 0.862 in the random effect model, whereas the specificity was 0.535 in the common effect model and 0.484 in the random effect model, indicating a low specificity in the latter model. The heterogeneity I^2^ of both models was 79.5% in sensitivity and 96.7% in specificity; thus, it is reasonable to converge to the random effect model (Figure 1).

### Bivariate analysis using one cut-off within a study

#### Calculation of summary statistics

The reitsma function from the “mada” package, which is suitable for a bivariate model, was used. In the reitsma function, 13 data extracted from a single cut-off value with the highest Youden index for each study were entered, and because calculation is not possible if there is ‘0’ in a data cell, 0.5 was entered into all cells of the study (correction.control= “all”) or only the cells of the corresponding study (horizontal) (correction.control=“single”) were corrected to prevent error in the calculation. In the options, it is possible to adjust to an arbitrary value such as “correction= 0.5,” where 0.5 is the default value. For models using the reitsma function, “fit” was assigned.

**Figure 2.**
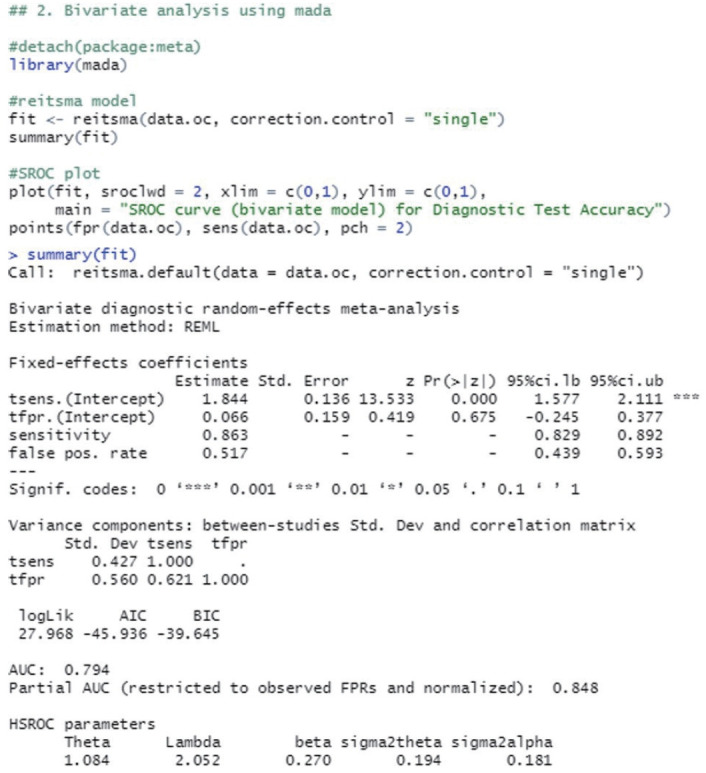
Bivariate analysis using one cut-off within a study (mada package).

Examining the result values through summary(fit), the values of sensitivity 0.863 and specificity 0.483 (= 1−0.517 FP rate) were obtained. The area under the curve (AUC) value of 0.794 can be observed in the middle of the console window, and values corresponding to the HSROC model are also shown (Figure 2).

The SROC curve uses the object calculated with the reitsma function. When a graph is drawn on the order of a command, the SROC curve first drawn with the “plot” function is temporarily saved in the computer memory, so individual studies are additionally indicated with the “points” function.

**Figure 3.**
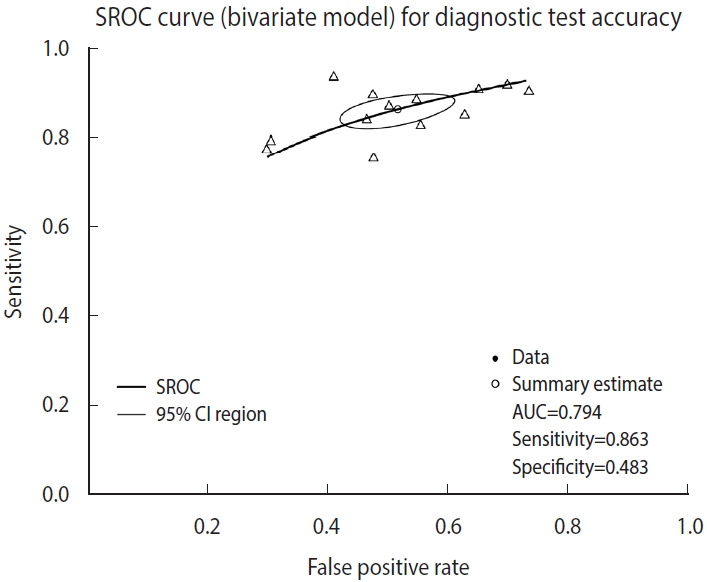
Summary receiver operating characteristic (SROC) curve (bivariate analysis using one cut-off within a study) for diagnostic test accuracy. CI, confidence interval; AUC, area under the curve.

The function “plot” draws graphs. In the function, the set model was entered into “fit,” and “sroclwd= 2” represented the thickness of the SROC curve. The units of the x and y axes were adjusted by adjusting “xlim” and “ylim”, respectively. The current graph shows the range from a minimum of 0 to a maximum of 1. The information of individual studies is entered in “points.” “fpr()” and “sens()” respectively represent the FP rate and sensitivity of individual studies, and “pch= 2” indicates the shape. Different numbers indicate different shapes as follows: rectangle (0), circle (1), triangle (2), cross (3), scissors (4), rhombus (5), inverted triangle (6), star (8), and black dot (20). The shape that allows the best discrimination was selected considering the visibility (Figure 3).

#### Testing of heterogeneity

The summary statistics and the SROC summary line described above are the deliverables that must be presented for DTA studies. In the subsequent analysis, the heterogeneity of individual studies is tested in the same way as the general pairwise meta-analysis, and if there is any significant factor, it should be tested and reported.

The basic assumption of the SROC curve is that the shape of the ROC curve is identical in all studies. However, this basic assumption cannot hold if there is heterogeneity between studies.

There are a variety of causes of heterogeneity such as chance, difference in cut-off value, difference in study design, prevalence, study environment, and the demographic factors of the sample population [1,4]. In meta-analysis of DTA studies, there are various methods for testing and diagnosing the status of heterogeneity [1,4]. In the first method, the asymmetry of the SROC curve is verified. In the second one, the possibility of heterogeneity is suspected if the degree of scattering, that is, the variation of individual studies in the SROC curve, is large. In the third method, heterogeneity may be suspected if the between-study variation is greater than the within-study variation in the forest plot (sensitivity, specificity, diagnostic odds ratio [DOR]). These methods only depend on visual discrimination, and therefore, the researchers should be able to observe the overall outline and formulate a comprehensive judgement on the heterogeneity.

The symmetry of the SROC curve indicates the agreement between the models of a divided SROC curve when this curve is divided by an arbitrary line from the top of the y-axis to the bottom right of the x-axis. That is, a diagnostic test with high accuracy will show that the SROC curve is symmetrical, the inflection point is drawn to the top left corner, and the curve will have a sharp turn to increase its AUC. Then, the value of Youden’s J index (J=sensitivity+specificity−1) will increase.

According to the judgment based on visual discrimination, the SROC curve in this example does not appear to have a high asymmetry and the degree of scattering of individual studies also does not seem to be large. However, since the specificity is low compared to the high sensitivity, the inflection point of the entire curve is located in the center and top right instead of top left, which indicates that the diagnostic test does not have a sufficiently high accuracy. However, as the example diagnostic test is a test for diagnosing prostate cancer, a major chronic disease, through biomarkers, the abovementioned characteristic of the curve is acceptable from the point of view that the test is more specialized in the sensitivity for identifying the diseased. The specificity of the test can be improved through additional biopsy or radiological examination afterward, and thus the above example may be considered acceptable as the benefit of finding more immediate risk or threat to life (sensitivity) is greater than the benefit of accurately judging that the person is not a patient with a prostate cancer (specificity).

### Bivariate analysis using multiple cut-offs within a study

#### Calculation of summary statistics

The diagmeta function from the “diagmeta” package was used for the calculation. In the bivariate model of the “mada” package, which was considered above, 13 data extracted from a single cut-off value with the highest Youden index for each study were entered, but in the case of the diagmeta function, 34 data corresponding to all cut-off values were used. It is not necessary to match the number of cut-offs for each study.

The variable names in the example data of this study (Table 1) were changed to the same names of the parameters in the diagmeta function using the colnames function (“author”, “study_id”, “cutpoint”, “tpos”, “fpos”, “fneg”, “tneg”). In general, when functions are developed in a package, the variable names in the data are set to allow linking. However, sometimes there are functions in which the variable names cannot be linked and therefore, we recommend using the parameter names that are provided by default to reduce possible errors.

This model considered continuous biomarkers of the observed diseased group and the non-diseased group. In individual studies, when TP, FP, FN, and TN corresponding to each cut-off are given according to a random number of cut-offs, sensitivity and specificity can be calculated for the respective studies. In particular, this model was developed in accordance with the specificity and 1-sensitivity corresponding to the negative test result in the diagnostic test.


(1) $$h\left(SP\left(x\right)\right)=\frac{x-\mu 0}{\sigma 0},\;h\left(1-Se\left(x\right)\right)=\frac{x-\mu 1}{\sigma 1}$$

where h denotes the normal model or logistic model, Sp is the specificity, Se is the sensitivity, x is the threshold, and denote the mean and variance for non-diseased, and and represent the mean and variance for diseased.

From individual data, a normal distribution or a logistic model is used to derive the corresponding model (h) (1), and this model is transformed to a linear mixed effects model for fitting (2) and (3). In general, a linear mixed effects model includes fixed effects (α0, α1, β0, and β1) and random effects (a0, a1, b0, and b1), and the structure of the model is changed according to how the weights of these parameters are set, which correspond to the intercepts and slopes for the respective cases.


(2) $$h\left(\frac{TNsi}{n0s}\right)=\alpha 0+a0s+\left(\beta 0+b0s\right)xsi+esi$$

(3) $$h\left(\frac{FNsi}{n1s}\right)=\alpha 1+a1s+\left(\beta 1+b1s\right)xsi+fsi$$

where TN is true negative, FN is false negative, s denotes study, i is the threshold of the study, α0 and α1, β0 and β1 are fixed intercepts and fixed slopes for non-diseased and diseased, respectively; a0 and a1, b0 and b1 are random intercepts and random slopes for non-diseased and diseased, respectively; *e* is residual error for non-diseased, and f is residual error for diseased.

**Figure 4.**
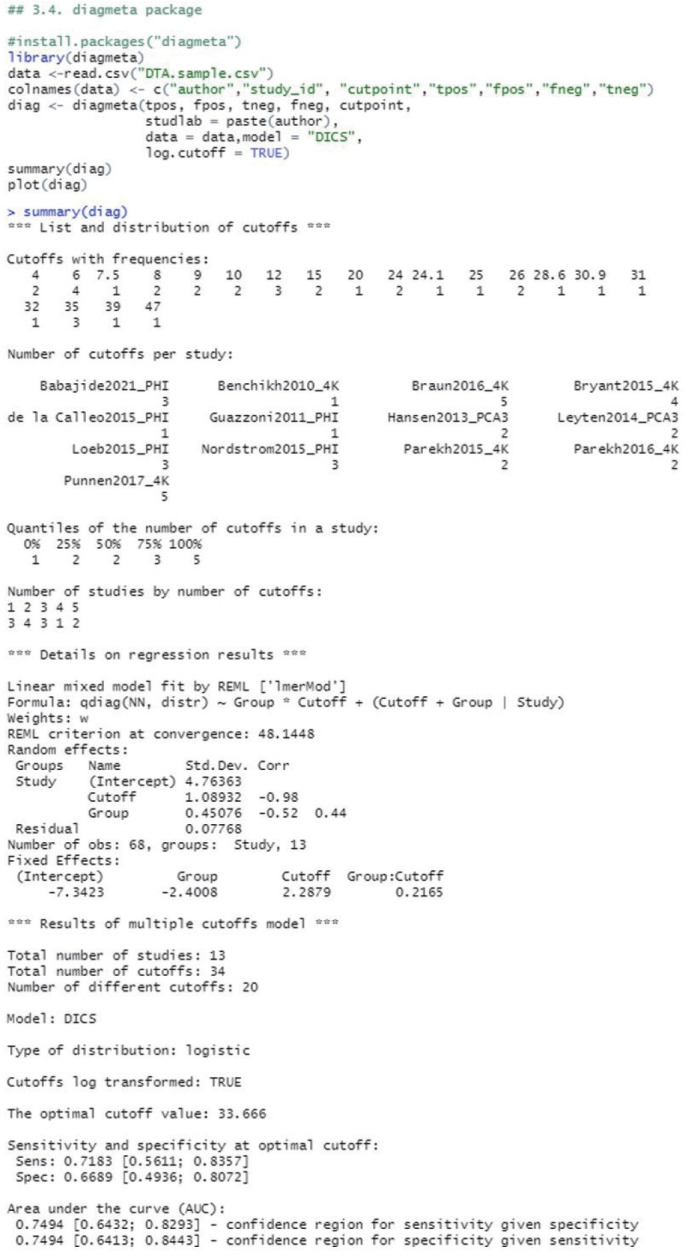
Bivariate analysis using multiple cut-offs within a study (diagmeta package).

As for the model of the diagmeta function, the weights of the individual intercept and slopes are selected when calculating the linear mixed effects model. For the selection, eight options of distribution function are given as follows: “DIDS”, “CIDS”, “DICS”, “CICS”, “DS”, “CS”, “DI”, and “CI” (Figure 4). For example, if it is assumed that the intercepts of individual studies are different and the slopes within the study are the same, the different random intercepts and common random slope (DICS), b0s= b1s= bs, function is selected [17,20,21]. Therefore, it is recommended that clinical researchers make a comprehensive judgment by comparing the values calculated using multiple models rather than trying to understand the problem of setting a statistical model in excessive detail.

“log.cutoff” sets whether to use the respective cutoffs after log transformation or use them as the raw data. In general, as the number of data is not large, it is necessary to normalize the values through log transformation.

Examining the results through the summary(diag) function, we can observe and verify the frequency of the cut-offs in detail as well as the number of cut-offs for individual studies. At the bottom of the result window, the total number of studies (13) and the number of used cut-offs (34) are shown as a summary, and the DICS function was used as the model. The optimal cut-off value was 33.666, sensitivity was 0.718, specificity was 0.669, and AUC value was 0.749 (Figure 4).

**Figure 5.**
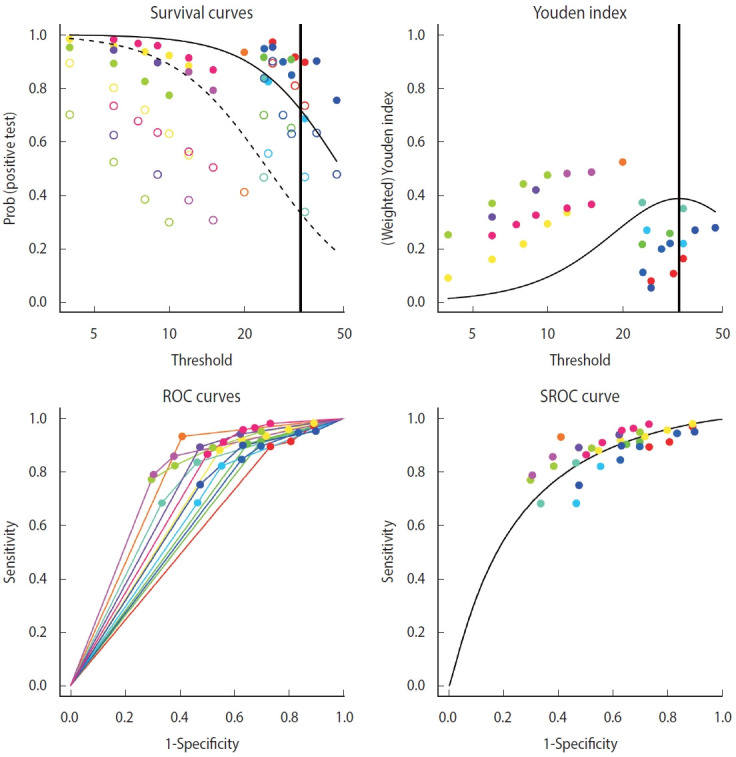
Summary receiver operating characteristic (SROC) curve (bivariate analysis using multiple cut-offs within a study) for diagnostic test accuracy.

Along with summary statistics, figures for deriving the statistics are presented (Figure 5). In the original paper on the development of the diagmeta package [17], survival curves were represented in terms of negative test results, but as the package development was already completed and the package was formally registered, the curves are now represented using positive test results. That is, sensitivity and 1-specificity according to the cut-off are presented using a scatter plot, and the diseased and non-diseased groups are indicated using solid and dashed lines, respectively. As for Youden index, the maximum value of the corresponding function was calculated and used as the optimal cut-off. The ROC curves for individual studies were plotted, and the points corresponding to sensitivity and 1-specificity were added by cut-off in the ROC curve of individual studies. Finally, a single SROC curve was created using a mixed linear regression model summarizing the individual ROC curves.

#### Analysis of heterogeneity

Comparing the bivariate model of the “mada” package with the SROC curve, the bivariate model of the “diagmeta” package, which has larger number of data by using all cut-offs, shows a better distribution in terms of heterogeneity. That is, the distribution of all studies was symmetrical and concentrated, showing no significant heterogeneity. In addition, as described above, as the distribution of all effect sizes is located at the top right corner compared to the SROC inflection point (0.718, 0.669), it can be observed that this example test is a diagnostic test specialized for sensitivity, showing relatively low specificity.

### Comparative analysis on the methods of meta-analysis of diagnostic test accuracy studies

In this work, for meta-analysis of DTA studies, summary statistics were calculated and the summary line was analyzed using the “meta”, “mada”, and “diagmeta” packages (Table 2). The summary statistics of the random effect model univariate analysis of the “meta” package with a single cut-off corresponding to the highest Youden index in a single study and those of the bivariate analysis of the “mada” package were highly similar. However, in the bivariate analysis of the “diagmeta” package including all cut-off values, the sensitivity decreased and the specificity increased as the amount of data increased. Considering the heterogeneity of the SROC curve and the use of all given cut-offs, the use of the bivariate analysis model of the “diagmeta” package is recommended.

Indeed, the “diagmeta” model also shows some limitations as follows. First, the model does not take into account the uncertainty of variance in individual studies, so there may be a problem of continuity correction. In particular, if there is “0” in a data cell in the 2× 2 table, calculation is not possible. Therefore, in this case, data preprocessing needs to be considered, such as the method of augmentation in which an identical set value (e.g., 0.5) is entered in each cell. Second, if there is excessive heterogeneity in the cutoff values, the fit may be poor. However, given that this problem is unavoidable owing to the nature of meta-analysis, it is necessary to perform cross-validation using various tools for meta-analysis of the DTA studies discussed above.

## CONCLUSION

In this study, I aimed to present the minimal theory of statistics and concentrate on the practical methods of meta-analysis of DTA studies for data integration so that researchers who are non-statistics majors can also perform the analyses with ease. Through this study, it is expected that researchers will be able to utilize the ready-made statistical tools that have been already developed and interpret them appropriately for each field of their research.

In particular, for systematic review and meta-analysis of DTA studies, when there are multiple effect sizes due to two or more cut-off values (thresholds), I presented a method that allows the use of all information without omission and a comparative analysis with the existing method. Therefore, the results of this study will provide researchers with a useful guideline for selecting an appropriate model for their studies.

In addition, it is expected that the findings of this study will facilitate the process of meta-analysis and promote related studies for many researchers in the relevant fields in Korea.

## Figures and Tables

**Table 1.** Data sample for diagnostic test accuracy

Name	Study	Cut-off	TP	FP	FN	TN	Biomarker	Youden	Y.c
Babajide2021_PHI	1	26	51	95	1	11	PHI	0.08	0
Babajide2021_PHI	1	32	48	86	4	20	PHI	0.11	0
Babajide2021_PHI	1	35	47	78	5	28	PHI	0.17	1
Benchikh2010_4K	2	20	175	333	12	480	4K	0.53	1
Braun2016_4K	3	4	206	707	3	84	4K	0.09	0
Braun2016_4K	3	6	200	634	8	158	4K	0.16	0
Braun2016_4K	3	8	195	569	13	223	4K	0.22	0
Braun2016_4K	3	10	192	498	16	294	4K	0.29	0
Braun2016_4K	3	12	184	434	24	358	4K	0.34	1
Bryant2015_4K	4	4	127	607	6	260	4K	0.25	0
Bryant2015_4K	4	6	119	453	14	414	4K	0.37	0
Bryant2015_4K	4	8	110	332	23	535	4K	0.44	0
Bryant2015_4K	4	10	103	258	30	609	4K	0.48	1
de la Calleo2015_PHI	5	24	112	191	10	82	PHI	0.22	1
Guazzoni2011_PHI	6	30.9	237	58	24	31	PHI	0.26	1
Hansen2013_PCA3	7	35	94	186	43	369	PCA3	0.35	0
Hansen2013_PCA3	7	24	115	258	22	297	PCA3	0.37	1
Leyten2014_PCA3	8	35	79	153	36	175	PCA3	0.22	0
Leyten2014_PCA3	8	25	95	182	20	146	PCA3	0.27	1
Loeb2015_PHI	9	31	136	301	24	178	PHI	0.22	1
Loeb2015_PHI	9	28.6	144	335	16	144	PHI	0.20	0
Loeb2015_PHI	9	24.1	152	401	8	78	PHI	0.11	0
Nordstrom^2^015_PHI	10	26	254	661	12	73	PHI	0.05	0
Nordstrom^2^015_PHI	10	39	240	464	26	270	PHI	0.27	0
Nordstrom^2^015_PHI	10	47	200	350	65	385	PHI	0.28	1
Parekh2015_4K	11	6	218	487	13	294	4K	0.32	0
Parekh2015_4K	11	9	207	371	24	410	4K	0.42	1
Parekh2016_4K	12	12	199	300	32	491	4K	0.48	0
Parekh2016_4K	12	15	183	238	48	543	4K	0.49	1
Punnen2017_4K	13	6	131	171	2	62	4K	0.25	0
Punnen2017_4K	13	7.5	127	159	4	76	4K	0.29	0
Punnen2017_4K	13	9	126	149	5	86	4K	0.33	0
Punnen2017_4K	13	12	120	132	11	103	4K	0.35	0
Punnen2017_4K	13	15	114	118	17	117	4K	0.37	1

TP, true positive; FP, false positive; FN, false negative; TN, true negative; Y.c, Youden index choice within a study; PHI, prostate health index; PCA, prostate cancer; 4K, 4-kallikrein.

**Table 2.** Summary statistics comparison by diagnostic test accuracy (DTA) methods

DTA methods	One cut-off within a study^1^			Multiple cut-offs within a study
	Univariate analysis		Bivariate analysis	Bivariate analysis
	Common model using “meta”	Random model using “meta”	“Mada”	“Diagmeta”
Sensitivity	0.844	0.862	0.863	0.718
Specificity	0.535	0.484	0.483	0.669

1 According to the high Youden index.

## SUPPLEMENTARY MATERIALS

Korean version is available at https://www.e-epih.org/.
