# Supplementary material for: Meta-analysis of diagnostic test accuracy studies with multiple thresholds for data integration
Source: Epidemiol Health. 2022 Sep 28;44:e2022083. doi: 10.4178/epih.e2022083 (PMC10106543; doi:10.4178/epih.e2022083)
Supplement: Supplementary file 1 [file epih-44-e2022083-suppl.pdf]

Comparison of diagnostic test accuracy modeling of meta-analysis for data integration

Sung Ryul Shim, Ph.D., M.P.H.

Department of Health and Medical Informatics, Kyungnam University College of Health Sciences, Changwon, Korea.

Correspondence

Sung Ryul Shim, Ph.D., M.P.H.

Department of Health and Medical Informatics, Kyungnam University College of Health Sciences, 7 Kyungnamdaehak-ro Masanhappo-gu, Changwon-si Gyeongsangnam-do 51767, Korea. Tel: +82-55-249-2729, Fax: +82-505-182-8972, Email: sungryul.shim@gmail.com

Epub ahead of print

Comparison of diagnostic test accuracy modeling of meta-analysis for data integration

Abstract

The objective of this study is to introduce how to use all of these information without omission when individual studies provide multiple effect sizes according to multiple cut-off values (thresholds) during diagnostic test meta-analysis for data integration. For diagnostic test meta-analysis, a general performance method of synthesizing data according to one cut value in one study and a performance method of synthesizing data according to two or more cut values in one study were compared and analyzed. In addition, this study focuses on the practical methods of DTA rather than theoretical concepts for researchers whose fields of study were non-statistics related. Through this, it is expected that general researchers will be able to use the already developed statistical tools to write and interpret them appropriately for their research fields. By performing this study, we hope that many researchers will use R software to determine the DTA more easily, and that there will be greater interest in related research.

Key words

Evidence based medicine, Meta-analysis, Systematic review, Research synthesize, Diagnostic tests, Diagnostic accuracy study, Receiver-operating characteristic curve, Threshold, ROC curve

Epub ahead of print

INTRODUCTION

본 연구는 진단검사 메타분석시 개별 대상연구가 다수의 기준치 (cut-off value, threshold)에 따른 여러 개의 효과크기를 제공하고 있을 때 누락없이 이들 정보를 모두 사용하는 방법을 소개한다. 따라서 기본적인 진단검사 메타분석에 대한 이해와 더불어 요약추정치 계산을 위한 통계모형은 선행연구들을 [1-4] 참고하여 반드시 사전에 지식을 습득하여야 한다.

일반적인 메타분석(pairwise meta-analysis)에서는 하나의 효과크기(effect size)를 산출하는데 반해서 진단검사 메타분석(diagnostic test accuracy)에서는 동시에 두 가지 짝지은 효과크기를 종합하는데 예를 들어 민감도(sensitivity)와 특이도(specificity)의 추정치를 동시에 계산한다. 이때 진단검사 메타분석은 기본적으로 대상 연구들의 기준치가 유사하다고 가정하지만 실제 대상연구들의 기준치는 동일하지 않고 다양하며 이것은 SROC(summary receiver operating characteristic) 곡선내 이질성의 주요 원인이 되기도 한다 [1].

Epub ahead of print

다수의 체계적 문헌고찰 연구들에서 진단검사 메타분석시 두개 이상의 기준치에 따른 효과크기 계산 방법을 다루었는데 [5-9], 이들은 하나의 연구내 ROC 커브에서 '민감도 + 특이도 - 1'이라는 매우 단순한 계산식을 통해 특이도와 민감도의 합이 최대가 되는 점을 구하는 Youden index를 통해서 가장 높은 하나의 기준치를 선택한 후 이변량 (bivariate) 모형을 사용하였으며 선택되지 않은 나머지 기준치들의 수치는 버려졌다. 이처럼 여러 개의 기준치에 따른 효과크기들이 있음에도 불구하고 하나만을 선택하게 된다면, 불완전보고 (incomplete reporting)를 스스로 선택하는 모순이 생기며 결국 모든 정보를 활용하지 못하게 된다.

이러한 문제점을 해결하고자 많은 연구자들이 진단검사 메타분석시 여러 개의 기준치에 따른 효과크기를 모두 사용하는 방법을 개발하였는데 [10-17], 이들 중 질병그룹(diseased group)과 비질병그룹(non-diseased group)각각의 분포함수를 활용하여 최적의 기준치와 전체 요약추정치인 민감도, 특이도, 그리고 SROC 곡선을 추정하는 방법을 소개하고자 한다 [17].

MATERIALS AND METHODS

1. 진단검사 요약추정치와 모형

진단검사 메타분석은 true positive(TP, 진양성), false positive(FP, 위양성), false negative(FN, 위음성), true negative(TN, 진음성)의 기본적인 2*2형태의 테이블로부터 요약추정치(summary statistics)와 요약곡선(summary line)으로 보여주게 된다. 일반적인 요약추정치로서는 민감도, 특이도, 양성예측도(PPV, positive predictive value), 그리고 음성예측도(NPV, negative predictive value) 등이 있으며 이러한 요약추정치를 종합한 요약곡선으로는 SROC가 대표적이다.

Epub ahead of print

진단검사 메타분석에서 요약추정치를 계산하기 위해서는 일반적인 메타분석과 마찬가지로 적합한 모형이 선택되어져야 하며 민감도와 특이도를 동시에 고려한 모형으로 대표적인 것이 이변량(bivariate) 모형과 그리고 계층적(hierarchical) 모형이다. 이변량 모형은 연구 내 변동은 민감도와 특이도를 직접 모형화한 이항분포(binominal distribution)를 가정하고 연구 간 변동은 이변량 정규분포(bivariate normal distribution)를 가정하는 반면 계층적 모형은 연구 내 변동은 이항분포를 가정하고 연구 간 변동은 이항분포의 확률에 대해 로지스틱 회귀모형을 적용하여 로지스틱 모형에 포함된 모수들에 대해 계층적 분포를 가정한다는 것이 다른 점이라고 할 수

있다 [1,4]. 그러나 두 모형 모두 일반 메타분석에서의 변량효과(random effect) 모형과 유사하며 공변량이 없을 때 수리적으로 두 모형은 동일한 값을 제공한다 [18,19].

본 논문에서는 R 소프트웨어의 “meta”,“mada”, “diagmeta”패키지를 통해서 값들을 추정할 것이기 때문에 관련 패키지를 미리 설치한다.

·install.packages("meta")

·install.packages("mada")

·install.packages("diagmeta")

2. 데이터 코딩 및 불러오기

진단검사 메타분석 예제자료 (Table 1)로서 전립선암 진단검사 연구 13개 (전체 기준치 포함 34개의 효과크기)를 수집하였으며, biomarker는 각 검사별 진단지표이고 cutoff는 기준치를 나타낸다. Youden은 민감도+특이도-1로 계산되어졌으며, 동일 연구내에 기준치가 2개 이상일 경우 Y.c가 가장 높은 것을 1로 코딩하였다.

meta와 mada 함수를 사용한 단변량 및 이변량 분석에서는 하나의 연구 내에서 Youden index가 가장 높은 하나의 기준치를 선택하여 분석하였으며, diagmeta 함수를 이용한 이변량분석에서는 모든 기준치를 포함하여 분석하였으며 최적의 기준치도 추정하였다.

RESULTS

1. 단변량 분석 (univariate analysis)

Epub ahead of print

"meta" 패키지 중 metaprop 함수는 비율형 자료에서 사건수(event)과 표본수(n)이 있을 때 종합 효과크기를 계산한다.

민감도 분석에서의 사건발생수는 TP이며 표본수는 TP + FN이다. 비율형 자료에서 효과크기를 계산하는 방식 중 로짓변환 후 환원하는 계산방법을 사용하였다 (Logit transformation과 Clopper-Pearson). 통계모형의 가정에 충실하고 자료의 대칭성과 분포를 감안했을 때 비율형 자료는 변환(로그변환 또는 로짓변환)하는 것이 보수적인 결과를 만들어내어 보다 바람직하다. Inverse variance method는 메타분석의 기본적인 방법으로서 개별 연구들의 가중치를 계산할 때 해당 연구의 역분산을 활용하며, DerSimonian-Lair estimator으로 Random effect model에서 연구간 변량인 tau값을 계산하였다.

민감도는 common effect model에서 0.844 그리고 random effect model에서 0.862를 나타내었고, 특이도는 common effect model에서 0.535 그리고 random effect model에서 0.484로서 random effect model에서 특이도가 낮게 계산되어졌다. 전체 모델의 이질성 I2가 민감도에서 79.5%, 특이도에서 96.7%로서 random effect model로 수렴하는 것이 타당하다 (Figure 1).

2. 연구별 하나의 기준치를 이용한 이변량 분석 (bivariate analysis using one cutoff within a study)

2.1 요약추정치 산출

Epub ahead of print

“mada” 패키지 중에서 이변량 모델에 적합한 reitsma 함수를 사용한다. reitsma 함수에 개별 연구별 Youden index가 가장 높은 하나의 기준치에서 추출한 13개의 데이터를 넣고, 데이터 셀이 '0'일 경우 계산불능 방지를 위해서 전체 연구의 모든 셀에 0.5를 넣거나 (correction.control = "all") 또는 해당연구(가로)의 셀들만 보정 (correction.control = "single")할 수 있다. 옵션에서 ‘correction = 0.5’ 처럼 임의 수치로 조절할 수도 있으며, 0.5가 default이다. reitsma 함수를 사용한 모델을 fit으로 객체 지정한다.

summary(fit)을 통해서 결과들을 살펴보면 민감도 0.863, 특이도 0.483(=1- 0.517 false positive rate)를 확인할 수 있다. 콘솔 창 중간 부분에 AUC값이 0.794을 표시하고 있으며 아울러 HSROC 모델에 해당하는 값들도 표시되니 참고하기 바란다 (Figure 2).

SROC 곡선은 reitsma 함수를 이용해서 산출한 객체를 이용한다. 그래프를 명령어 순서대로 그릴 때 컴퓨터메모리상 제일 처음 plot 함수로 그린 SROC 곡선이 임시저장 되기에 points 함수로 개별 연구는 추가로 표시된다.

plot은 그래프를 그리는 함수이다. 설정된 모델 fit을 넣고 sroclwd=2는 sroc곡선의 두께를 나타내며, xlim과 ylim을 조절해서 x,y축의 단위를 조절한다. 현재는 최소 0에서 최대 1까지를 표시한 것이다. points는 개별 연구를 넣어준다. fpr()과 sens()는 개별 연구들의 false positive rate와 sensitivity를 표시하는 것이며 pch=2은 모양을 나타낸다. 개별 모양은 네모(0), 동그라미(1), 세모(2), 십자가(3), 가위표(4), 마름모(5), 역삼각형(6), 별표(8), 검은점(20) 등 다양하며 가독성을 고려해서 식별력이 좋은 것으로 선택한다 (Figure 3).

Epub ahead of print

2.2 이질성검토

진단검사 메타분석에서 요약추정치와 SROC 요약곡선는 반드시 제시되어야 하는 산출물이다. 이후의 분석은 일반 메타분석과 동일하게 개별연구들의 이질성을 확인하고 만약 유의한 인자가 있다면 이를 검정하고 보고하여야 한다.

SROC 곡선의 기본 가정은 모든 연구에서 ROC 곡선의 형태가 동일하다는 것이다. 그렇지만, 연구들 간의 이질성이 존재한다면 이러한 기본 가정을 충족하지 못한다.

이질성의 원인은 매우 다양한데 우연(chance), 기준치 차이, 연구설계 차이, 유병률, 연구환경, 그리고 표본집단의 인구사회학적 요인 등을 들 수 있다 [1,4]. 진단검사 메타분석에서는 이런 이질성의 여부를 진단할 수 있는 다양한 방법들을 제시하고 있다 [1,4]. 첫째 SROC 곡선의 비대칭성, 둘째 SROC 곡선내 개별 연구들의 흩어짐 정도 즉 연구들의 변량이 크다면 이질성을 의심할 수 있으며, 마지막으로 Forest plot(민감도, 특이도, 진단오즈비)에서 연구 간 변동이 연구 내 변동보다 큰 경우 이질성을 의심할 수 있다. 모두 시각적인 구분에만 의존하기 때문에 연구자는 전체적인 윤곽을 보고 종합적인 판단을 할 수 있는 능력이 요구된다.

Epub ahead of print

SROC 곡선의 대칭성이란 SROC 곡선의 Y축 상단에서 X축 우하단으로 임의의 직선을 나누었을 때 양분 되어진 SROC 곡선의 모형이 일치하는지를 보는 것이다. 즉, 좋은 진단검사일수록 SROC 곡선이 대칭성을 나타내며 변곡점이 좌측 최상단에 나타나며, 곡선이 급격히 휘어짐으로서 SROC 곡선 아래면적(AUC, area under the curve)이 커지게 되고 Youden's J index(J = 민감도 + 특이도 – 1)가 높아진다고 가늠할 수 있다.

본 예제의 SROC 곡선은 육안으로 보기에 비대칭성은 크지 않을 것으로 판단되며 개별 연구들의

흩어짐의 정도도 크지 않아 보인다. 그러나 높은 민감도에 비해 낮은 특이도를 나타내고 있어 전체 곡선의 변곡점이 좌측 상단이 아닌 중앙과 우측상단으로 위치하고 있어 진단검사의 입장에서는 만족스러운 결과라고는 할 수 없을 것이다. 하지만 생체지표(biomarker)를 통해 중증질환인 전립선암을 진단하는 검사로서 질환자 확인을 위한 민감도에 보다 특화되었다는 관점에서는 충분히 받아들일 만하다. 이후의 추가적인 조직검사(biopsy)나 영상의학적 검사를 통해서 특이도를 보완할 수 있기때문에 당장의 혹시 있을지 모를 생명의 위해를 더 많이 찾아내는 편익이 (민감도), 질환자가 아님을 정확히 맞추는 편익 (특이도) 보다 더 클 수 있기 때문이다.

Epub ahead of print

3. 연구별 여러개의 기준치를 이용한 이변량 분석 (bivariate analysis using multiple cutoffs within a study)

3.1 요약추정치 산출

“diagmeta” 패키지의 diagmeta 함수를 사용한다. 앞서 실시한 “mada” 패키지의 이변량 모델은 Youden index가 가장 높은 연구당 하나의 기준치 13개의 데이터를 넣고 분석한 반면, diagmeta에서는 모든 기준치에 해당하는 데이터 34개를 사용한다. 연구별로 기준치의 개수가 반드시 같을 필요는 없다.

본 연구의 예제자료 (Table 1)의 변수명을 colnames 함수를 이용해서 diagmeta에서 사용하는 구성인자와 동일한 이름으로 변경하였다 ("author","study_id", "cutpoint","tpos","fpos","fneg","tneg").

일반적으로 패키지 내의 함수들이 개발될 때 데이터 내의 변수명을 연결할 수 있게 설정하고 있으나 그렇지 않은 함수들도 가끔씩 있기 때문에 시행착오를 줄이기 위해서 defalut로 사용되는 구성인자명을 사용하는 것을 추천한다.

본 모형은 관찰된 질병그룹 (diseased group)과 비질병그룹 (non-diseased group)의 연속형 biomarker를 고려하였는데, 개별 연구들에서 임의의 기준치 개수에 따라 각 기준치에 해당하는 TP, FP, FN, 그리고 TN이 주어질 때 각각의 민감도와 특이도를 계산할 수 있다. 특히 본 모형은 진단검사시 음성 (negative test result)에 해당하는 특이도와 1-민감도에 맞추어 개발되어졌다.

$$\mathrm{h}(\mathrm{Sp}(x)) = \frac{x-\mu 0}{\sigma 0},\ \mathrm{h}(1-\mathrm{Se}(x)) = \frac{x-\mu 1}{\sigma 1} \qquad \text{수식 (1)}$$

h, normal model or logistic model; Sp, specificity; Se, sensitivity; x, threshold; μ0 and σ0, mean and variance for non-diseased; μ1 and σ1, mean and variance for diseased

Epub ahead of print

개별 데이터로부터 정규분포 또는 로지스틱 모형을 이용하여 해당 모형 (h)를 구한다음 (수식 1) 이를 다시 선형혼합모형 (linear mixed effects model)로 변환하여 적합시킨다 (수식 2 & 3). 일반적으로 선형혼합모형은 고정효과 (fixed effects; α0, α1, β0 그리고 β1)와 랜덤효과 (random effects; a0, a1, b0 그리고 b1)를 포함하는데, 각 절편과 기울기에 해당하는 이러한 인자들의 가중치를 어떻게 설정하는가에 따라서 전체적인 모형의 구조가 바뀌어 진다.

$$\mathrm{h}\left(\frac{\mathrm{TN}si}{\mathrm{n}0s}\right) = \alpha 0 + \mathrm{a}0s + (\beta 0 + \mathrm{b}0s)xsi + esi \qquad \text{수식 (2)}$$

$$\mathrm{h}\left(\frac{\mathrm{FN}si}{\mathrm{n}1s}\right) = \alpha 1 + \mathrm{a}1s + (\beta 1 + \mathrm{b}1s)xsi + fsi \qquad \text{수식 (3)}$$

TN, true negative; FN, false negative; s, study; i, threshold of the study; α0 and α1, β0 and β1, fixed intercepts and fixed slopes for non-diseased and diseased; a0 and a1, b0 and b1, random intercepts and random slopes for non-diseased and

diseased; e, residual error for non-diseased; f, residual error for diseased

diagmeta 함수의 model은 선형혼합모형 계산시에 절편(intercept)과 기울기(slope) 개별 인자들의 가중치를 선택하는 것으로 "DIDS", "CIDS", "DICS", "CICS", "DS", "CS", "DI", "CI" 으로 8개의 분포함수 옵션이 주어진다 (Figure 4). 예를 들어 개별 연구들의 절편은 다르며 연구내에서의 기울기는 동일할 것으로 가정한다면 DICS (Different random intercepts and common random slope, b0s=b1s=bs)을 설정한다 [17, 20, 21]. 따라서 임상 연구자들은 통계모형의 설정문제에 대해 너무 깊이 있는 이해보다는 여러개의 모형을 통해서 산출된 값을 상호비교 해보면서 종합적인 판단을 하는 것을 권고한다.

log.cutoff는 각 기준치를 로그변환으로 사용할 것인지 그렇지 않으면 원자료 그대로 사용할 것인가를 설정하는 것인데, 일반적으로 데이터의 개수가 많지 않기 때문에 로그변환을 통해서 조금 더 정규화하는 것이 필요하다.

summary(diag)을 통해서 결과들을 살펴보면 개별 연구별 기준치의 개수와 더불어 기준치의 빈도를 상세하게 확인할 수 있다. 결과창 하단에 보면 전체 연구수 13개, 사용된 기준치 34개를 정리해서 보여주며 모델은 DICS를 사용했다. 최적의 기준치는 33.666이었으며, 민감도 0.718, 특이도 0.669, 그리고 AUC값 0.749를 나타내었다 (Figure 4).

요약추정치와 더불어 이를 수렴해가는 과정의 관련 그림들을 나타내었다 (Figure 5). Survival curves는 원래 diagmeta 개발논문 [17]에서 negative test result로 표시하고 있었는데 패키지 개발이 완료되고 등재된 이후는 positive test result로 변경된 것으로 보인다. 즉, 기준치에 따른 민감도

Epub ahead of print

와 1-특이도를 산점도로 표시하고 질병그룹(diseased group, solid line)과 비질병그룹(non-diseased group, dashed line)를 표시한 것이다. Youden index에서는 해당 함수의 최고점을 계산하여 최적의 기준치로 수렴하였다. ROC curves는 개별 연구별 ROC를 도식화하고 개별 연구의 ROC 내에 기준치별로 민감도와 1-특이도에 해당하는 포인트를 추가하였다. 최종적으로 SROC curve에서 개별 ROC들을 종합한 mixed linear regression model을 통해서 하나의 SROC를 만들었다.

3.2 이질성검토

“mada” 패키지의 이변량 모델과 SROC 요약곡선을 비교해서 보면 모든 기준치를 사용함으로서 데이터의 양이 풍부해진 “diagmeta” 패키지의 이변량 모델이 더욱 좋은 분포를 나타낸다. 즉, 전체 연구들의 분포가 대칭적이고 집중되어 있어 큰 이질성은 찾을 수 없었다. 더불어 앞서 설명한 바와 같이 전체 효과크기들의 분포가 SROC 변곡점 (0.718, 0.669) 보다 우상단으로 위치해 있어 민감도에 특화된 진단검사로서, 상대적으로 낮은 특이도를 보여주는 결과라는 것을 알 수 있다.

Epub ahead of print

4. 진담검사 메타분석 수행기법별 비교 분석

지금까지 진단검사 메타분석을 위해서“meta”, “mada”, 그리고 “diagmeta”패키지를 이용한 요약추정치 산출과 요약곡선을 분석해 보았다. 하나의 연구 내에서 Youden index가 가장 높은 하나의 기준치를 선택한 “meta”패키지의 Random effect model 단변량분석과 “mada”의 이변량분석의 요

약추정치는 매우 유사하였다. 그러나 모든 기준치를 포함한 "diagmeta"패키지의 이변량분석에서는 데이터의 양이 많아지면서 민감도는 감소하고, 특이도는 증가하는 경향을 보였다. SROC 요약곡선의 이질성을 고려하고 주어진 모든 기준치 데이터 사용을 고려한다면"diagmeta"의 이변량분석 모델이 보다 바람직한 방향으로 추정된다.

물론 "diagmeta" 모델도 몇 가지 제한점을 포함하는데 첫번째, 개별 연구단위에서의 분산에 대한 불확도 (uncertainty)를 고려하지 않았기에 연속성보정 (continuity correction)에 대한 문제가 발생할 수 있으며 특히 2*2 테이블에서 0을 포함한 셀이 있을 경우 계산불능에 이른다. 따라서 이럴 경우 각 셀에 동일한 수치 (예, 0.5)를 강제로 넣어 주는 증강방법 (augment) 등으로 전처리를 고려해주어야 한다. 두번째, 기준치들이 너무 이질 할 경우 적합도가 떨어질 수 있다. 그러나 이것은 메타분석의 속성상 불가피한 것이기 때문에 앞서 살펴본 다양한 진단검사 메타분석 도구들을 사용하여 교차 검증하는 것이 필요하다.

Epub ahead of print

CONCLUSIONS

본 연구는 통계학을 전공하지 않은 일반 연구자들도 쉽게 수행할 수 있도록 통계학 이론은 최소화하고 데이터통합을 위한 진단검사 메타분석의 실질적 수행방법에 집중하였다. 이를 통해서 일반 연구자들이 이미 개발되어진 통계 도구들을 잘 활용하여 본인의 연구분야에 적합하게 쓰고 해석할 수 있을 것으로 기대한다.

특히 체계적 문헌고찰 및 진단검사 메타분석시 두 개 이상의 기준치에 따른 여러 효과크기를 제시한다면, 정보의 누락없이 이를 모두 사용하는 방법과 더불어 기존의 수행방법도 비교 분석하였

기에 본인의 연구에 적합한 모델 선택에 도움이 될 것이다.

아울러 본 연구를 통하여 많은 국내 연구자들이 보다 쉽게 메타분석을 수행하고 관련 연구가 활성화되기를 바란다.

Acknowledgment

Funding

This work was supported by Kyungnam University Foundation Grant, 2022 (No. 2022-0034)

Conflict of Interest Disclosures

The author of this manuscript declare no relationships with any interest related to the subject matter of the article.

Author Contributions

Shim SR had full access to all of the data in the study and take responsibility for the integrity of the data and the accuracy of the data analysis.

Concept and design, Acquisition, analysis, or interpretation of data, Drafting of the manuscript, Critical revision of the manuscript for important intellectual content, Statistical analysis: Shim SR

Epub ahead of print

References

[1] Shim SR, Kim SJ. Lee JH. Diagnostic test accuracy: application and practice using R software Epidemiol Health. 2019;41:e2019007.

[2] 심성률, 의학보건학 연구자를 위한 R 메타분석, 한나래출판사, 2019.

[3] Higgins JPT, Green S (editors). Cochrane Handbook for Systematic Reviews of Interventions. Version 5.1.0 [updated March 2011]. The Cochrane Collaboration, 2011. Available from http://www.cochrane-handbook.org. [accessed on 28 Sept 2021]

[4] 심성률, 신인수, 배종면. "STATA를 이용한 진단검사 정확도 메타분석". 한국보건정보통계학회지 40.3 (2015): 190-199.

[5] Roberts E, Ludman AJ, Dworzynski K, Al-Mohammad A, Cowie MR, McMurray JJ, Mant J. The diagnostic accuracy of the natriuretic peptides in heart failure: systematic review and diagnostic meta-analysis in the acute care setting. Br Med J. 2015;350:910. on behalf of the NICE Guideline Development Group for Acute Heart Failure.

[6]. Aertgeerts B, Buntinx F, Kester A. The value of the CAGE in screening for alcohol abuse and alcohol dependence in general clinical populations: a diagnostic meta-analysis. J Clin Epidemiol. 2004;57(1):30–9.

[7] Zhelev Z, Hyde C, Youngman E, Rogers M, Fleming S, Slade T, Coelho H, Jones-Hughes TVN. Diagnostic accuracy of single baseline measurement of elecsys troponin T high-sensitive assay for diagnosis of acute myocardial infarction in emergency department: systematic review and meta-analysis. BMJ. 2015;350:15. doi:10.1136/bmj.h15.

[8] Wacker C, Prkno A, Brunkhorst FM, Schlattmann P. Procalcitonin as a diagnostic marker for sepsis: a systematic review and meta-analysis. Lancet Infect Dis. 2013;13(5):426–35.

[9] Vouloumanou EK, Plessa E, Karageorgopoulos DE, Mantadakis E, Falagas ME. Serum procalcitonin as a diagnostic marker for neonatal sepsis: a systematic review and meta-analysis. Intensive Care Med. 2011;37(5):747–62.

[10] Hamza TH, Arends LR, van Houwelingen HC, Stijnen T. Multivariate random effects meta-analysis of diagnostic tests with multiple thresholds. BMC Med Res Methodol. 2009;10(9):73.

[11] Leeflang MM, Deeks JJ, Takwoingi Y, Macaskill P. Cochrane diagnostic test accuracy reviews. Syst Rev. 2013;2:82. doi:10.1186/2046-4053-2-82.

[12] Dukic V, Gatsonis C. Meta-analysis of diagnostic test accuracy assessment studies with varying number of thresholds. Biometrics. 2003;59:936–46.

[13] Putter H, Fiocco M, Stijnen T. Meta-analysis of diagnostic test accuracy studies with multiple

Epub ahead of print

thresholds using survival methods. Biometrical J. 2010;52(1):95–110.

[14] Martínez-Camblor P. Fully non-parametric receiver operating characteristic curve estimation for random-effects meta-analysis. Stat Methods Med Res. 2014. doi:10.1177/0962280214537047.

[15] Riley R, Takwoingi Y, Trikalinos T, Guha A, Biswas A, Ensor J, Morris RK, Deeks J. Meta-analysis of test accuracy studies with multiple and missing thresholds: a multivariate-normal model. J Biometrics Biostat. 2014;5:196.10.4172/2155-6180.1000196.

[16] Riley RD, Elia EG, Malin G, Hemming K, Price MP. Multivariate meta-analysis of prognostic factor studies with multiple cut-points and/or methods of measurement. Stat Med. 2015;34(17):2481–96.

[17] Steinhauser S, Schumacher M, Rücker G. Modelling multiple thresholds in meta-analysis of diagnostic test accuracy studies. BMC Med Res Methodol. 2016 Aug 12;16(1):97. doi: 10.1186/s12874-016-0196-1. PMID: 27520527; PMCID: PMC4983029.[5] Smeeth L, Haines A, Ebrahim S. Numbers needed to treat derived from meta-analyses--sometimes informative, usually misleading. BMJ 1999; 318(7197): 1548-1551.

[18] Arends LR, Hamza TH, van Houwelingen JC, Heijenbrok Kal MH, Hunink MG, Stijnen T. Bivariate random effects meta analysis of ROC curves. Medical Decision Making 2008;28(5):621 638.

[19] Harbord RM, Deeks JJ, Egger M, Whiting P, Sterne JA. A unification of models for metaanalysis of diagnostic accuracy studies. Biostatistics 2007;8(2):239 251.

[20] Gerta Rücker, Susanne Steinhauser, Srinath Kolampally, Guido Schwarzer. Meta-Analysis of Diagnostic Accuracy Studies with Several Cutpoints; 2022 [cited 2022 April 22]. Available from: https://cran.r-project.org/web/packages/diagmeta/diagmeta.pdf

[21] Guido Schwarzer. Official Git repository for R package diagmeta - GitHub. Available from: https://github.com/guido-s/diagmeta

Epub ahead of print

Table 1. Data sample for diagnostic test accuracy.

name	study	cutoff	TP	FP	FN	TN	Biomarker	Youden	Y.c
Babajide2021_PHI	1	26	51	95	1	11	PHI	0.08	0
Babajide2021_PHI	1	32	48	86	4	20	PHI	0.11	0
Babajide2021_PHI	1	35	47	78	5	28	PHI	0.17	1
Benchikh2010_4K	2	20	175	333	12	480	4K	0.53	1
Braun2016_4K	3	4	206	707	3	84	4K	0.09	0
Braun2016_4K	3	6	200	634	8	158	4K	0.16	0
Braun2016_4K	3	8	195	569	13	223	4K	0.22	0
Braun2016_4K	3	10	192	498	16	294	4K	0.29	0
Braun2016_4K	3	12	184	434	24	358	4K	0.34	1
Bryant2015_4K	4	4	127	607	6	260	4K	0.25	0
Bryant2015_4K	4	6	119	453	14	414	4K	0.37	0
Bryant2015_4K	4	8	110	332	23	535	4K	0.44	0
Bryant2015_4K	4	10	103	258	30	609	4K	0.48	1
de la Calleo2015_PHI	5	24	112	191	10	82	PHI	0.22	1
Guazzoni2011_PHI	6	30.9	237	58	24	31	PHI	0.26	1
Hansen2013_PCA3	7	35	94	186	43	369	PCA3	0.35	0
Hansen2013_PCA3	7	24	115	258	22	297	PCA3	0.37	1
Leyten2014_PCA3	8	35	79	153	36	175	PCA3	0.22	0
Leyten2014_PCA3	8	25	95	182	20	146	PCA3	0.27	1
Loeb2015_PHI	9	31	136	301	24	178	PHI	0.22	1
Loeb2015_PHI	9	28.6	144	335	16	144	PHI	0.20	0
Loeb2015_PHI	9	24.1	152	401	8	78	PHI	0.11	0
Nordstrom2015_PHI	10	26	254	661	12	73	PHI	0.05	0
Nordstrom2015_PHI	10	39	240	464	26	270	PHI	0.27	0
Nordstrom2015_PHI	10	47	200	350	65	385	PHI	0.28	1
Parekh2015_4K	11	6	218	487	13	294	4K	0.32	0
Parekh2015_4K	11	9	207	371	24	410	4K	0.42	1
Parekh2016_4K	12	12	199	300	32	491	4K	0.48	0
Parekh2016_4K	12	15	183	238	48	543	4K	0.49	1
Punnen2017_4K	13	6	131	171	2	62	4K	0.25	0
Punnen2017_4K	13	7.5	127	159	4	76	4K	0.29	0
Punnen2017_4K	13	9	126	149	5	86	4K	0.33	0
Punnen2017_4K	13	12	120	132	11	103	4K	0.35	0
Punnen2017_4K	13	15	114	118	17	117	4K	0.37	1

Y.c, Youden index choice within a study.

Epub ahead of print

Table 2. Summary statistics comparison by diagnostic test accuracy methods

DTA methods	One cutoff within a study			Multiple cutoffs within a study
	Univariate analysis		Bivariate analysis	Bivariate analysis
	Common model using “meta”	Random model using “meta”	“mada”	“diagmeta”
Sensitivity	0.844	0.862	0.863	0.718
Specificity	0.535	0.484	0.483	0.669

one cutoff within a study according to the high Youden index

Epub ahead of print

Figure legends

Figure 1. Univariate analysis using meta package.

Figure 2. Bivariate analysis using one cutoff within a study (mada package).

Figure 3. Summary receiver operating characteristic (SROC) curve (bivariate analysis using one cutoff within a study) for diagnostic test accuracy. CI, confidence interval; AUC, area under the curve.

Figure 4. Bivariate analysis using multiple cutoffs within a study (diagmeta package).

Figure 5. Summary receiver operating characteristic (SROC) curve (bivariate analysis using multiple cutoffs within a study) for diagnostic test accuracy.

Epub ahead of print

```
#Data open
data <-read.csv("DTA.sample.csv")
#One data obtained from one cutoff by Youden index high value within a study
data.oc <- data[data$Y.c==1,]
#All data obtained from multiple cutoffs within a study
data.mc <- data

## 1. Univariate analysis using meta

library(meta)
#Sensitivity
sensitivity <- metaprop(TP, TP+FN,comb.fixed=T,comb.random= TRUE,sm="PLOGIT",
method = "Inverse", method.ci="CP",studlab=name, byvar=Biomarker, data=data.oc)
print(sensitivity, digits=3)

#Specificity
specificity <- metaprop(TN, TN+FP,comb.fixed=T,comb.random= TRUE,sm="PLOGIT",
method = "Inverse", method.ci="CP",studlab=name, byvar=Biomarker, data=data.oc)
print(specificity, digits=3)
> print(sensitivity, digits=3)
Number of studies combined: k = 13
Number of observations: o = 2233
Number of events: e = 1908

                     proportion          95%-CI
Common effect model       0.844 [0.828; 0.859]
Random effects model      0.862 [0.827; 0.890]
> print(specificity, digits=3)
Number of studies combined: k = 13
Number of observations: o = 6834
Number of events: e = 3664

                     proportion          95%-CI
Common effect model       0.535 [0.523; 0.547]
Random effects model      0.484 [0.407; 0.561]
```

Epub ahead of print

```
## 2. Bivariate analysis using mada

#detach(package:meta)
library(mada)

#reitsma model
fit <- reitsma(data.oc, correction.control = "single")
summary(fit)

#SROC plot
plot(fit, sroclwd = 2, xlim = c(0,1), ylim = c(0,1),
     main = "SROC curve (bivariate model) for Diagnostic Test Accuracy")
points(fpr(data.oc), sens(data.oc), pch = 2)
```

```
> summary(fit)
Call:  reitsma.default(data = data.oc, correction.control = "single")

Bivariate diagnostic random-effects meta-analysis
Estimation method: REML

Fixed-effects coefficients
                  Estimate Std. Error      z Pr(>|z|) 95%ci.lb 95%ci.ub
tsens.(Intercept)    1.844      0.136 13.533    0.000    1.577    2.111 ***
tfpr.(Intercept)     0.066      0.159  0.419    0.675   -0.245    0.377
sensitivity          0.863          -      -        -    0.829    0.892
false pos. rate      0.517          -      -        -    0.439    0.593
---
Signif. codes:  0 '***' 0.001 '**' 0.01 '*' 0.05 '.' 0.1 ' ' 1

Variance components: between-studies Std. Dev and correlation matrix
      Std. Dev tsens  tfpr
tsens    0.427 1.000     .
tfpr     0.560 0.621 1.000

 logLik     AIC     BIC
 27.968 -45.936 -39.645

AUC:  0.794
Partial AUC (restricted to observed FPRs and normalized):  0.848

HSROC parameters
      Theta      Lambda        beta sigma2theta sigma2alpha
      1.084       2.052       0.270       0.194       0.181
```

Epub ahead of print

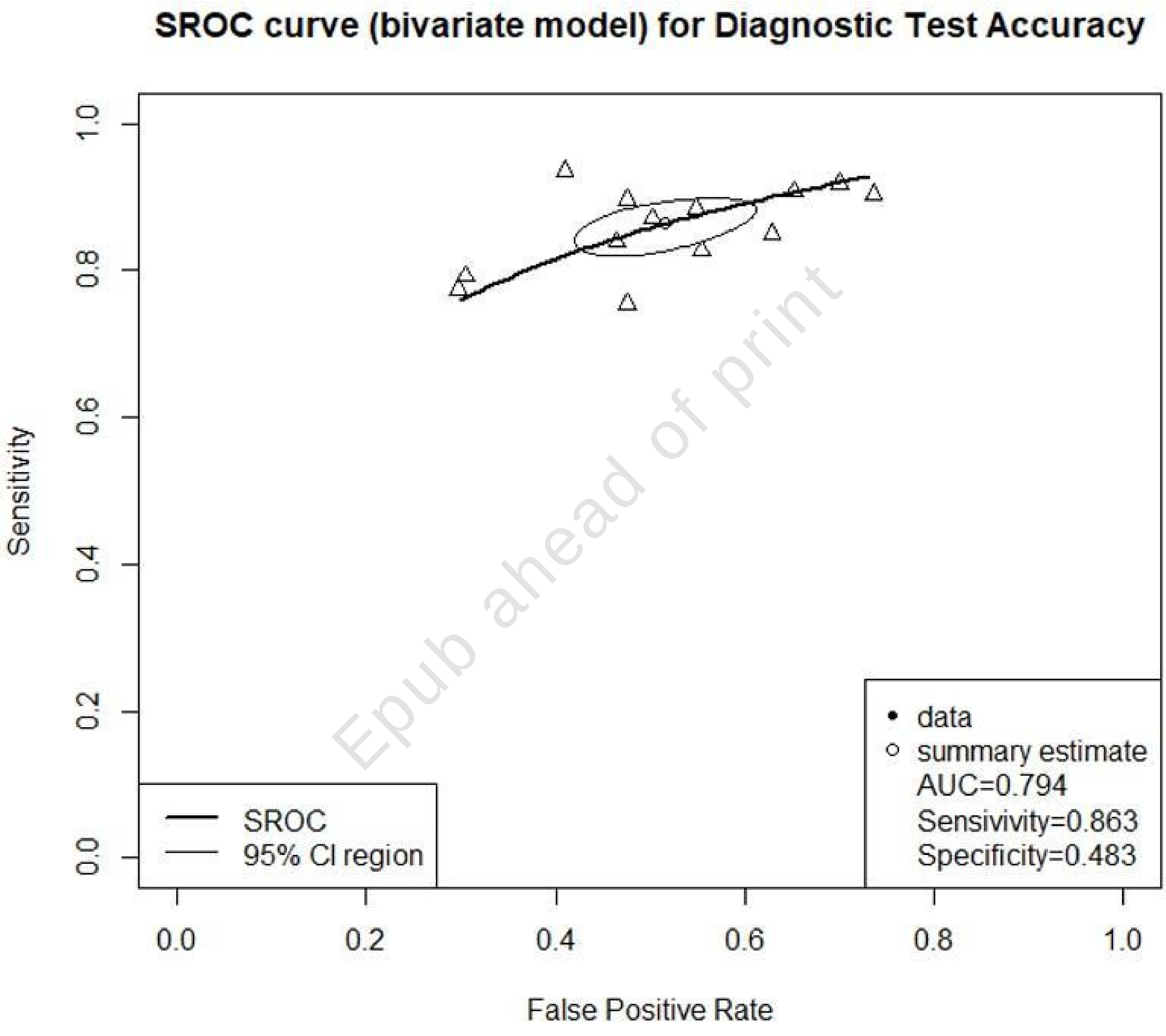

Epub ahead of print

```r
## 3.4. diagmeta package

#install.packages("diagmeta")
library(diagmeta)
data <-read.csv("DTA.sample.csv")
colnames(data) <- c("author","study_id", "cutpoint","tpos","fpos","fneg","tneg")
diag <- diagmeta(tpos, fpos, tneg, fneg, cutpoint,
                 studlab = paste(author),
                 data = data,model = "DICS",
                 log.cutoff = TRUE)
summary(diag)
plot(diag)
```

```
> summary(diag)
*** List and distribution of cutoffs ***

Cutoffs with frequencies:
   4    6  7.5    8    9   10   12   15   20   24 24.1   25   26 28.6 30.9   31
   2    4    1    2    2    2    3    2    1    2    1    1    2    1    1    1
  32   35   39   47
   1    3    1    1

Number of cutoffs per study:

    Babajide2021_PHI      Benchikh2010_4K          Braun2016_4K         Bryant2015_4K
                   3                    1                     5                     4
de la Calleo2015_PHI     Guazzoni2011_PHI        Hansen2013_PCA3       Leyten2014_PCA3
                   1                    1                     2                     2
        Loeb2015_PHI     Nordstrom2015_PHI         Parekh2015_4K         Parekh2016_4K
                   3                    3                     2                     2
       Punnen2017_4K
                   5

Quantiles of the number of cutoffs in a study:
  0%  25%  50%  75% 100%
   1    2    2    3    5

Number of studies by number of cutoffs:
1 2 3 4 5
3 4 3 1 2

*** Details on regression results ***

Linear mixed model fit by REML ['lmerMod']
Formula: qdiag(NN, distr) ~ Group * Cutoff + (Cutoff + Group | Study)
Weights: w
REML criterion at convergence: 48.1448
Random effects:
 Groups   Name        Std.Dev. Corr
 Study    (Intercept) 4.76363
          Cutoff      1.08932  -0.98
          Group       0.45076  -0.52  0.44
 Residual             0.07768
Number of obs: 68, groups:  Study, 13
Fixed Effects:
 (Intercept)        Group       Cutoff  Group:Cutoff
     -7.3423      -2.4008       2.2879        0.2165

*** Results of multiple cutoffs model ***

Total number of studies: 13
Total number of cutoffs: 34
Number of different cutoffs: 20

Model: DICS

Type of distribution: logistic

Cutoffs log transformed: TRUE

The optimal cutoff value: 33.666

Sensitivity and specificity at optimal cutoff:
 Sens: 0.7183 [0.5611; 0.8357]
 Spec: 0.6689 [0.4936; 0.8072]

Area under the curve (AUC):
 0.7494 [0.6432; 0.8293] - confidence region for sensitivity given specificity
 0.7494 [0.6413; 0.8443] - confidence region for specificity given sensitivity
```

Epub ahead of print

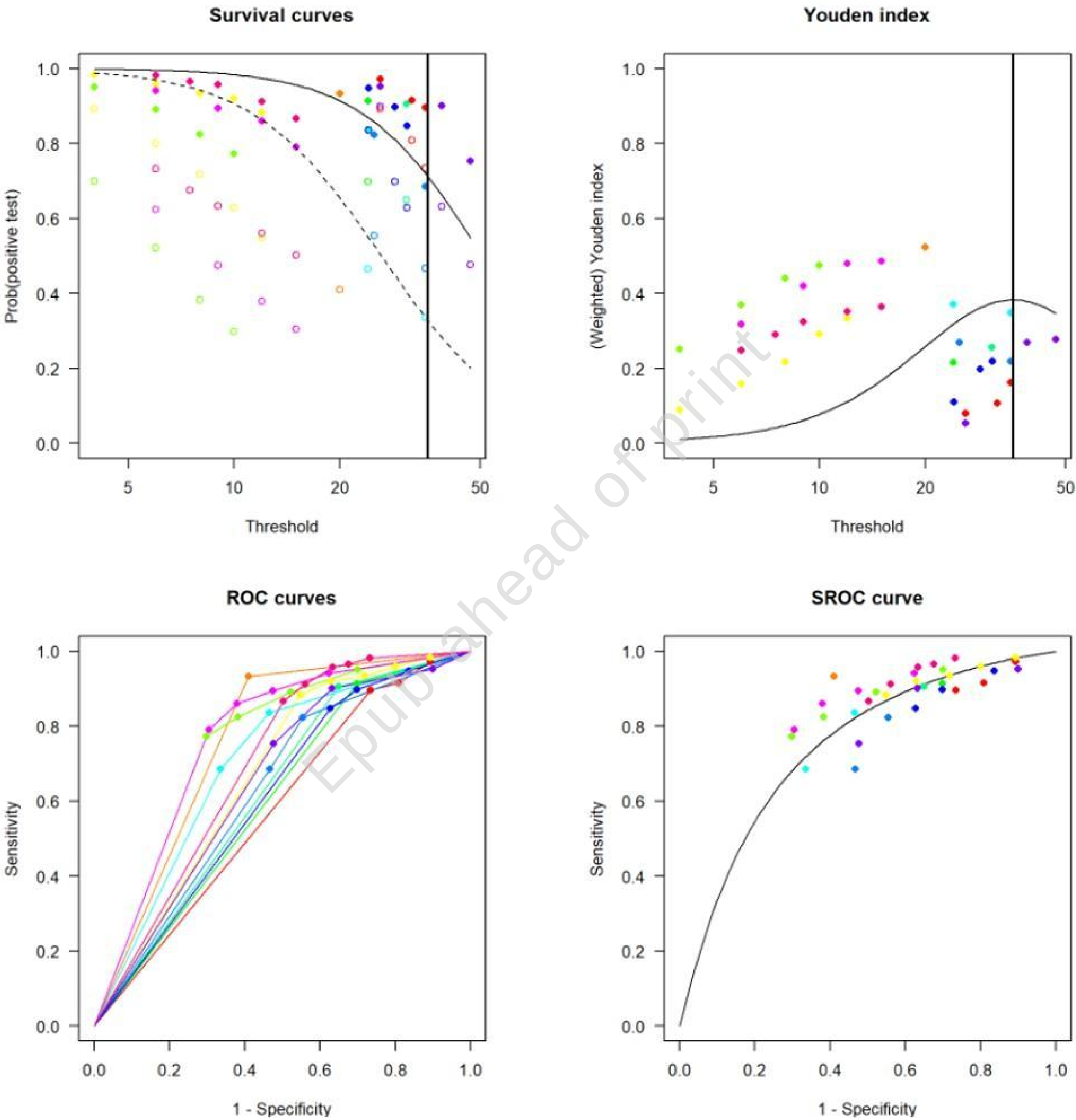

Epub ahead of print
